# Attachment of *L. ferrooxidans* to Pyrite Mineral Surfaces

**DOI:** 10.3390/microorganisms14010040

**Published:** 2025-12-23

**Authors:** Sian M. La Vars, Benjamin Watts, Jamie S. Quinton, Sarah L. Harmer

**Affiliations:** 1 Flinders Microscopy and Microanalysis, College of Science and Engineering, Flinders University, GPO Box 2100, Adelaide 5001, Australiaj.quinton@massey.ac.nz (J.S.Q.); 2 Photon Science Division, Paul Scherrer Institute (PSI), 5232 Villigen, Switzerland; benjamin.watts@psi.ch; 3 Flinders Institute for Nanoscale Science and Technology, College of Science and Engineering, Flinders University, GPO Box 2100, Adelaide 5001, Australia; 4 School of Food Technology and Natural Sciences, Massey University, Private Bag 11222, Palmerston North 4442, New Zealand

**Keywords:** ToF-SIMS, *Leptospirillum ferrooxidans*, pyrite, bioflotation, hydrophobicity

## Abstract

*L. ferrooxidans* and their metabolic products have been explored as viable flotation reagents of pyrite and chalcopyrite for froth flotation. Scanning electron microscopy (SEM), near edge X-ray absorption fine structure (NEXAFS) spectroscopy, time-of-flight secondary ion mass spectrometry (ToF-SIMS) and captive bubble contact angle measurements have been used to examine the surface physicochemical properties of pyrite upon exposure to *L. ferrooxidans* grown in HH medium at pH 1.8. C K-edge NEXAFS spectra, collected using scanning transmission X-ray microscopy (STXM), indicate hydrophilic lipids, fatty acids, and biopolymers are formed at the mineral–bacterium interface within hours of exposure. The Fe L-edge NEXAFS show oxidation of the mineral surface from Fe (II) sulfide to Fe (III) oxyhydroxides. The leaching of the iron species at the pyrite surface is accelerated in the presence of *L. ferrooxidans* and extracellular polymeric substances (EPS) as compared to HH medium controls, as shown by ToF-SIMS. The surface chemical changes induced by the interaction with *L. ferrooxidans* show a significant decrease in surface hydrophobicity within the first 2 h of exposure. The implications of these findings are the potential use of EPS, produced during early attachment of *L. ferrooxidans*, as a depressant for bioflotation or to enhance bioleaching.

## 1. Introduction

The sulfide mineral chalcopyrite (CuFeS_2_) is the world’s primary source of copper [1,2]. Copper is used in plumbing, electronic products, car components, and building construction, which makes chalcopyrite a desirable commercial mineral [1,2,3]. Pyrite (FeS_2_) is also a sulfide mineral, and although it can be used as a source of sulfur, it is generally considered an undesirable gangue mineral that is often found in ores along with the copper-containing chalcopyrite [2,4]. In order to extract the copper from chalcopyrite, traditional froth flotation uses chemical reagents to selectively alter a mineral’s propensity for repelling or attracting water; however, there are major health and environmental issues surrounding many of these reagents [5,6].

In bioflotation, microorganisms and their extracellular polymeric substances (EPS) are used instead of chemicals to improve the separation of minerals by acting as flotation reagents to alter the surface charge, hydrophobicity, and surface chemistry of the minerals [6,7,8,9]. Microorganisms can selectively interact with the gangue and the desired minerals, and can also be used in conjunction with chemical flotation reagents [10,11]. It is common for bioflotation investigations to include pre-concentrating the microorganisms or the addition of synthetic flotation reagents to cultures, which has been shown to negatively impact cell activity [12,13,14,15].

Bacteria that have been most commonly and effectively used for bioflotation of sulfide minerals are *Acidithiobacillus ferrooxidans*, *Leptospirillum ferrooxidans*, and *Acidithiobacillus thiooxidans*. These bacteria have been shown to affect the surface chemistry of sulfide minerals and as such are excellent reagents for the alteration of the flotation properties of the minerals due to their ability to adhere to and/or oxidize mineral surfaces [9,16]. *L. ferrooxidans*, though able to oxidize iron, have not been as extensively investigated as *A. ferrooxidans* in terms of bioflotation, but are commonly investigated in terms of bioleaching and often combined with other strains [17,18,19]. *L. ferrooxidans* is a Gram-negative bacterium that was first reported by Markosyan in 1972 [20]. This bacterium appears as a curved rod between 1 and 3.3 µm in length, obtains nutrients solely from the oxidation of ferrous iron, and grows ideally in aerobic, mesophilic, and acidic conditions [20].

*L. ferrooxidans* have previously been applied to bioleaching studies and have the advantage of being able to withstand slightly higher temperatures than *A. ferrooxidans*, although still within the mesophilic temperature range of 20–40 °C [17,21,22,23,24,25,26,27,28]. This strain is comparable in growth activity to *A. ferrooxidans* within the mesophilic temperature range; however, Sand et al. has determined that its iron-oxidizing activity is not as strong when grown on soluble iron (II) [18]. Harneit et al. has demonstrated that *L. ferrooxidans* have an affinity for pyrite; however, they noted that there were differences in cell behavior between strains and that there were complications in analyzing EPS components due to hydrolysis [29].

Unlike the bacterium *A. ferrooxidans* investigated previously [30], *L. ferrooxidans* is unable to use sulfur as a source of nutrients, potentially creating a different chemical environment on the surface of sulfide minerals [17,20,31]. This bacterial strain is widely recognized as a major producer of EPS, which would suggest it would be ideal for flotation studies, where large amounts of biomass are required for industrial applications [17,23,32]. It has also been identified as a more efficient oxidizer of pyrite than *A. ferrooxidans*, suggesting any surface effects due to oxidation on the surface may be accelerated in comparison [33].

The pure strain of this microorganism is currently not as commonly used for bioflotation, although many studies use it as part of mixed cultures, leaving its application as a flotation reagent largely unexplored [22,23,24,25,27]. Bioflotation investigations using *L. ferrooxidans* have focused mainly on the mineral recovery of chalcopyrite, sphalerite, pyrrhotite, and pyrite, with no investigation of the cells or the sulfide mineral with inconsistent conditions and often contradictory findings making comparison challenging [8,34,35]. These studies perform flotation experiments using a chemical collector, which has been shown to negatively impact cell activity, and thus is likely to interfere with mineral–cell interactions and EPS production [13]. They also involve the processing of the cells, including washing and artificially concentrating the cells by centrifugation before exposing the cells to the mineral which may be altering natural cell behavior and changing the nature of their excretions [8,34,35].

A thorough study of a pure strain of *L. ferrooxidans*, in the absence of synthetic flotation reagent and other forms of pre-processing, for its potential application to bioflotation has yet to be explored for pyrite–chalcopyrite separation. In this study, pyrite is exposed to *L. ferrooxidans* and the surface of the mineral will be analyzed for signs of EPS and bacterial attachment, with special interest taken in the time-frame over which attachment occurs and how the chemical species on the surface are changed by the presence of the bacteria. No previous study has been found on the bioleaching or bioflotation capabilities of *L. ferrooxidans* that successfully compares mineral surface roughness in relation to the hydrophobicity of the sample and the presence and spread of cells on the surface.

## 2. Materials and Methods

### 2.1. Culturing Microorganisms

The bacterial strain *L. ferrooxidans* (DSM 2705) was purchased from DSMZ (Leibniz-Institut DSMZ-Deutsche Sammlung von Mikroorganism und Zellkulturen, Braunschweig, Germany) and grown on modified Leptospirillum (HH) medium, DSM medium 882. The medium consists of 0.132 g NH_4_SO_4_, 0.053 g MgCl_2_.6H_2_O, 0.027 g KH_2_PO_4_, CaCl_2_.2H_2_O in 1 L ultrapure water adjusted to pH 1.8 with H_2_SO_4_. An iron (II) sulfate heptahydrate solution is added to base cultures as a soluble energy source when mineral is not present. A total of 20 g of ground pyrite or chalcopyrite (+37 μm, −75 μm) was UV sterilized and added to sterile 500 mL conical flasks prior to inoculation by *L. ferrooxidans* at 10% inoculum. Cultures were grown in an orbital mixer shaken at 155 rpm at 30 °C, and continuously cultured every 4 days into fresh HH medium (5% inoculum) as a stock culture.

### 2.2. Pyrite Tile Preparation and Exposure to L. ferrooxidans

Cubic pyrite (FeS_2_) was cut to 1–2 mm thickness and 5 mm^2^ in area and polished using wet/dry sandpaper of increasingly fine grain size, then polished with 1 μm diamond paste. The tiles were sonicated for 3 min in ultrapure water and UV sterilized in a laminar flow hood prior to exposure experiments. *L. ferrooxidans* was inoculated at 10% inoculum into sterile HH medium containing the sterilized pyrite tiles with no additional iron source. The polished pyrite pieces were removed at 2, 24, 72, and 168 hrs and either analyzed immediately or snap frozen in sterile HH medium, stored at −80 °C, and kept frozen until ready for analysis.

### 2.3. Scanning Electron Microscopy (SEM)

Pyrite tiles were removed from the cultures and stored in 3% glutaraldehyde at 4 °C prior to dehydration. The tiles were rinsed by immersion in phosphate-buffered saline solution for 10 min and post-fixed by immersion in 1% osmium tetroxide for 30 min. The samples were then dehydrated by immersion in increasingly concentrated ethanol solutions for 10 min per rinse starting at 1 × 70% *v*/*v*, followed by 1 × 90% *v*/*v*, 1 × 95% *v*/*v*, and 2 × 100% *v*/*v*, with a final rinse in hexamethyl-disilazane (HMDS) for 30 min [36,37]. Samples were then air-dried and mounted to SEM stubs using carbon tape and sputter coated with 3 nm of platinum.

Samples were analyzed on an Inspect FEI F50 Scanning Electron Microscope (FEI, Hillsboro, OR, USA) with a field emission electron emitter and EDX, backscatter, and secondary electron (SE) detectors. The primary electron beam accelerating voltage of 10 kV was used in Secondary Electron (SE) mode.

### 2.4. Atomic Force Microscopy (AFM)

AFM measurements were carried out using a Multimode Nanoscope V in tapping mode over a 10 μm spot size with a minimum of 5 spots per sample. The collected images were analyzed for surface roughness using the NanoScope Analysis software, version 1.40. Images were flattened in the software, and only the most necessary image processing to remove scan lines was performed to maintain the integrity of the data. Surface roughness can be calculated in two different ways: *R_a_* and *R_q_. R_a_* is the mean surface roughness, which is the mean value of the surface relative to the center plane for which the dimensions above and below are equal. *R_a_* is calculated by Equation (1), where *N* is the number of data points for the given area and *Z_j_* is the surface relative to the center plane [38].

(1) $$R_{a}=\frac{1}{N}\sum_{j=1}^{N}\left|Z_{j}\right|$$

*R_q_* is the root mean square of the height (*Z*) data and represents the standard deviation of the *Z* values within a given area. *R_q_* is calculated by Equation (2), where *Z_i_* is the current value and *N* is the number of data points all for the given area [38].

(2) $$R_{q}=\sqrt{\frac{\sum {\left(Z_{i}\right)}^{2}}{N}}$$

Roughness values are dependent on not only sample preparation but also on the dimensions of the tip being used to scan the surface, so while they can be compared for similar samples, the calculated roughness values are not absolute.

### 2.5. Captive Bubble Contact Angle

Pyrite tiles were removed from culture flasks and placed in the sterile HH medium to prevent drying before being loaded directly into the quartz cell for analysis. The samples were held face down and the sample face was immersed in solution. A J-shaped needle was positioned under the sample, through which air was pumped onto the surface. The volume of the bubble was increased over a period of approximately 60 s, with an image of the bubble captured after every volume increase to capture the receding angles. The process was reversed to capture the advancing angles. Based on the captive bubble methods described in previous studies [39,40], the last 5 images taken before the bubble was removed from the surface were used for the advancing angle, and the central 5 images from the receding phase were used for the receding angle. Captive bubble experiments were performed using the Sinterface Profile Analysis Tensiometer PAT1 Version 8 (Sinterface, Berlin, Germany) and the contact angles from the images captured by the Sinterface PAT1 CCD camera were analyzed using “ImageJ” (v8) imaging analysis software [41].

### 2.6. Near Edge Absorption Fine Structure (NEXAFS)

NEXAFS spectra were collected on the PolLux Scanning Transmission X-ray Microscope beamline at the Paul Scherrer Institut (PSI) Swiss Light Source (SLS) [42,43]. Five mL aliquots of culture were inoculated at 10% inoculum in fresh medium with −38 µm size fraction pyrite or chalcopyrite, with samples taken at 2, 24, and 336 h. Culture samples were agitated and larger particles were allowed to settle slightly so that the finer particles (<5 µm) remained in the supernatant. A syringe was used to withdraw 1 mL of culture, and 1 drop was applied to a 100 nm thick silicon nitride window. Drops were reapplied as the previous drop dried to build a sparse layer of mineral particles over the surface. The windows were checked under an optical microscope to make sure the samples were not too thick, and to be sure the window remained unbroken.

Images were collected over an area of 20 µm under helium atmosphere with a gold zone plate with an outer most zone width of 35 nm. Image stacks were collected by scanning the energy range of 280–320 eV for the C *K*-edge, so that each pixel of the image contained a complete NEXAFS spectrum taken at 0.1 eV increments. Images and image stacks were converted from transmission to optical density (OD) and aligned using the aXis2000 software (version 11, May 2016) [44].

The alignment process ensures the x, y limits are the same for each image in the stack using a polynomial 2-D transformation to align successive images to 4 fiducial points on the first image. After alignment was performed, the spectra of regions of interest (ROIs) were exported. Conversion to OD was performed by dividing the image or spectrum by a spectrum collected on a blank window. NEXAFS spectra around the C K-edge and Fe *L*-edge within the energy ranges of 280–310 eV and 700–732 eV, respectively, were collected by line scan, and images were collected at specific energies within these ranges. Line scans are performed by scanning the sample along a single line drawn along the sample in an area of interest over the energy range of interest. The Fe L-edge spectra were calibrated to the Fe^II^ L_3_ peak of pyrite 708.3 eV [45,46]. The C K-edge spectra were calibrated to the DNA carbon standard at 285.1 eV [47,48]. All NEXAFS spectra were pre-edge and post-edge corrected by a linear pre- and post-edge fit using “Athena” (v1.0) software [49]. All spectra were normalized to the corrected pre- and post-edge regions.

### 2.7. Time of Flight–Secondary Ion Mass Spectrometry (ToF-SIMS)

ToF-SIMS is a surface sensitive technique that uses a pulsed ion beam to fragment ions from the first 1–2 atomic layers of a surface. The mass of those fragments is measured and provides elemental and chemical analysis of the surface. The ToF-SIMS experiments were performed using a Physical Electronics Inc. (Chanhassen, MN, USA) PHI TRIFT V nanoTOF instrument equipped with a pulsed liquid metal 79+Au primary ion gun (LMIG), operating at 30 kV energy. “Unbunched” beam settings were used to optimize spatial resolution. A cold stage was employed to prevent the loss of volatile species to the vacuum. A minimum temperature of −70 °C was reached and maintained for the duration of analysis. The mass spectra and images obtained by ToF-SIMS were calibrated using “WinCadenceN” software version 1.8.1.3 (PHI, Chanhassen, MN, USA). The mass spectra were calibrated to the CH_3_^+^, C_2_H_5_^+^, and C_3_H_7_^+^ peaks for positive ion mass spectra, and CH^−^, C_2_H^−^, and Cl^−^ peaks in the negative ion mass spectra. Both positive and negative spectra were collected over an area of 100 μm^2^, with a minimum of 5 areas collected per sample. Integrated peak values of the selected ions were normalized to the total selected secondary ion intensities to correct for differences in the total ion yield between samples. Statistical analysis was carried out using a Student’s t-distribution with 95% probability [50,51,52]. The results have been plotted using a 95% confidence interval and may be compared qualitatively.

## 3. Results

### 3.1. Cell Growth of Leptospirillum ferrooxidans on Pyrite

**Figure 1:**
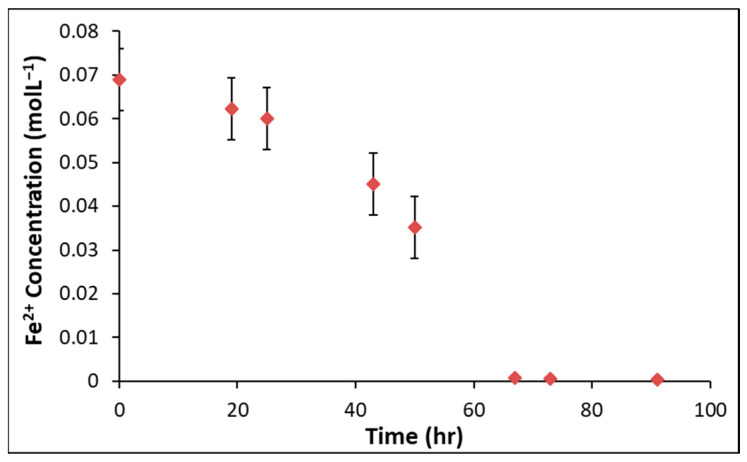
Ferrous iron titration curves of *L. ferrooxidans* in HH medium at 5% inoculum.

The live culture of *L. ferrooxidans* was first cultured on HH medium containing ferrous sulfate, with growth of this culture monitored by titrating against cerium (IV) sulfate to determine the concentration of Fe (II), from which the bacteria obtain their energy as it oxidizes to Fe (III) [53,54]. Figure 1 shows a typical concentration curve of the *L. ferrooxidans* base culture at 5% inoculum.

**Figure 2:**
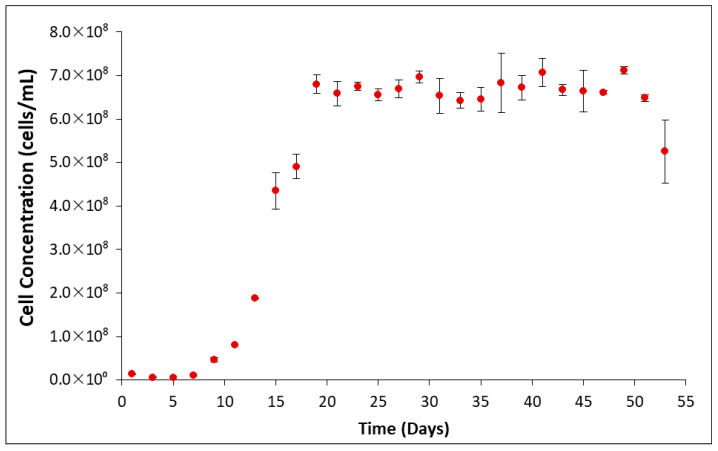
Typical growth curve of *L. ferrooxidans* on pyrite (+38, −75 um) at 10% inoculum.

The ferrous iron concentration in *L. ferrooxidans* grown on HH medium containing ferrous sulfate reaches exponential phase between 20 and 40 h, reaching the stationary phase after 72 h. Growth curves on pyrite were determined by counting using a hemocytometer at 40× magnification [28], and a typical growth curve is shown in Figure 2.

When grown on a solution that contains soluble ferrous iron, *L. ferrooxidans* typically reaches the exponential phase between 20 and 40 h, completing a growth cycle in approximately 96 h (Figure 1). When grown on pyrite, the *L. ferrooxidans* culture typically reaches the exponential phase between 11 and 19 days, completing a growth cycle in approximately 7 weeks (Figure 2). Final cell concentrations of *L. ferrooxidans* on pyrite were found to be over 6.5 × 10^8^ cells/mL.

### 3.2. Scanning Electron Microscopy

**Figure 3:**
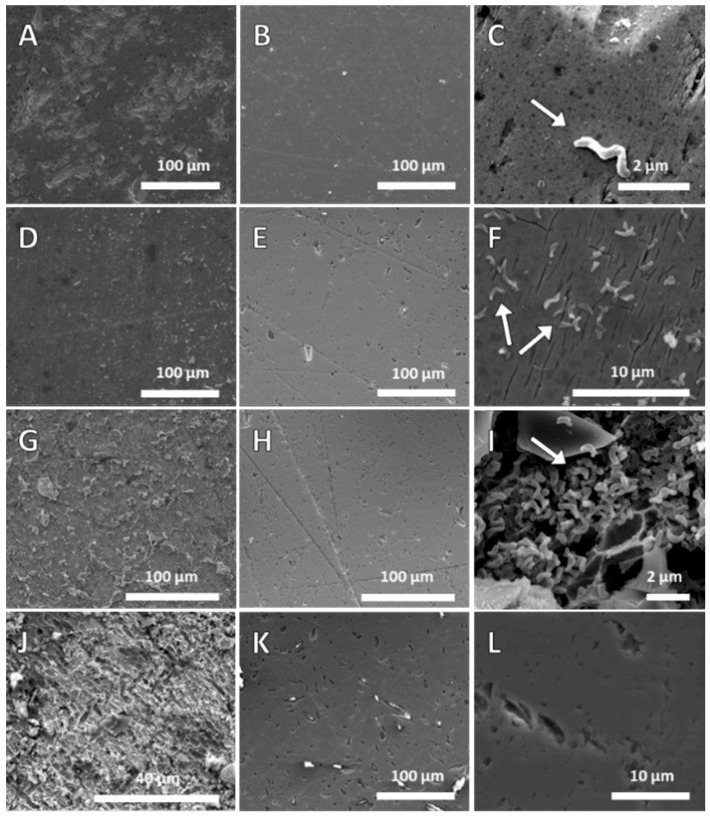
Pyrite tiles exposed to *L. ferrooxidans* for 2 h (**A**), 24 h (**D**), 72 h (**G**) and 168 h (**J**), pyrite tiles exposed to HH medium for 2 h (**B**), 24 h (**E**), 72 h (**H**), and 168 h (**K**), *L. ferrooxidans* cells, indicated by arrows at 2 h (**C**), leaching rivers after 24 h (**F**), *L. ferrooxidans* biofilm formation at 72 h (**I**), and etch pits at 168 h (**L**).

To relate solution conditions to the mineral leaching and cell interaction with the mineral surface, scanning electron microscopy (SEM) is used to provide images of the pyrite and *L. ferrooxidans*. Typical SEM images obtained for pyrite upon exposure to HH medium control in comparison to pyrite exposed to *L. ferrooxidans* are shown in Figure 3.

The surface of pyrite exposed to HH medium is shown in Figure 3B,E,H,K, the pyrite shows some pitting, is typically regularly orientated, and is 0.8–1.8 μm in length and 0.2–0.8 μm width, with some scratches remaining on the surface from the polishing process. Etch pits increased in number on the surface after 168 h. The pyrite exposed to the medium for 2 h is all but identical to the bare polished pyrite.

After 2 h exposure to pyrite (see Figure 3L), *L. ferrooxidans* cells are observed on the surface with no apparent preference for surface defects as cells were found on smooth areas of the surface (see Figure 3C). Etch pits on the surface are comparable in size to those observed on the control mineral at this period of exposure. *L. ferrooxidans* cells were found to be an average of 0.76 µm^2^ in area, with cell coverage at this stage of exposure calculated at 0.57 ± 0.14%. After 24 h of exposure of the pyrite surface to *L. ferrooxidans*, the cell population dramatically increased. There is still no apparent preference for surface defects of the cells on the surface, and the cells appear to be uniformly distributed across the surface with random orientation, shown in Figure 3F. There is a greater amount of loose debris on the surface compared with the HH medium control (Figure 3D). The sample exposed to the *L. ferrooxidans* also appears to have fine cracks appearing in the mineral surface (Figure 3F) [30]. The cell coverage at this stage of exposure has been calculated at approximately 3.32 ± 0.40%. After 72 h of exposure to *L. ferrooxidans*, the pyrite is covered in debris in a wide range of shapes and sizes, visible in Figure 3G. The surface is showing deeper rivers along crystal boundaries, rather than the cracks observed at 24 h. These rivers have been identified by previous investigations to be caused by ferric iron leaching and not direct bacterial action [55]. Cells cover the surface in random orientation and are also seen adhered to the mineral debris, with cell coverage measured at 10.7 ± 3.94%. This coverage is more than double the coverage observed at 24 h. After 168 h of exposure to *L. ferrooxidans*, the pyrite sample is heavily leached and covered in cells and biofilm, the extent of which is difficult to quantify due to the number of particles coated in cells that have adhered to the surface, as are observed in Figure 3I. The surface shows signs of advanced leaching (Figure 3J), with some areas of the surface exhibiting a charging due to the thick carbon layer from the biofilm coating the surface, making clear images hard to obtain in these areas. Advanced pitting in regularly arranged shapes in sizes ranging from 3 to 9 μm long and 2.5 to 3.5 μm wide cover the surface, and appear coated in strand-like material.

The results found here agree with previous studies, which suggest that this strain has been found to adhere more significantly to pyrite than other microorganisms [23] and dominates adhered cell populations over longer periods of exposure [28]. The number of cells on the surface is seen to increase, which agrees with many adhesion studies performed on *L. ferrooxidans* [23,28,34]. The amount of debris on the surface appears to be increasing at a significant rate, with etching of the surface appearing more advanced compared to the control. These results suggest initial attachment takes place prior to the significant mineral leaching observed in the Eh/pH and ICPOES, indicating cell attachment and/or EPS production is necessary for *L. ferrooxidans* to impact the pyrite surface. This further suggests either the direct contact mechanism, the indirect contact mechanism, or a combination of both determines the interaction of this strain with the pyrite surface.

Very few studies were identified as having performed abiotic control leaching experiments for analysis using SEM compared to biotic leaching experiments. Studies by Xia et al. and Mitsunobu et al. observed a smooth mineral surface on pyrite after extended periods of exposure (≥20 days), both of which agrees with the comparatively smooth control surfaces observed in this study [56,57]. Several studies air-dry their samples rather than preserving them with glutaraldehyde followed by dehydrating and fixing them, which makes any comparison to attachment of cells on the surface impossible [19,57]. The lack of control images being collected in biotic leaching studies, combined with the lack of consistency in sample preparation and pH of leaching solutions, makes comparing results challenging, and isolating bacterial action from chemical leaching more difficult. The information on the propagation of this microorganism on mineral surfaces provided by this study fills an important gap in the literature, showing an unexpected lack of preference of this strain for defect sites and the much-accelerated biofilm formation compared to other more heavily studied strains.

**Figure 4:**
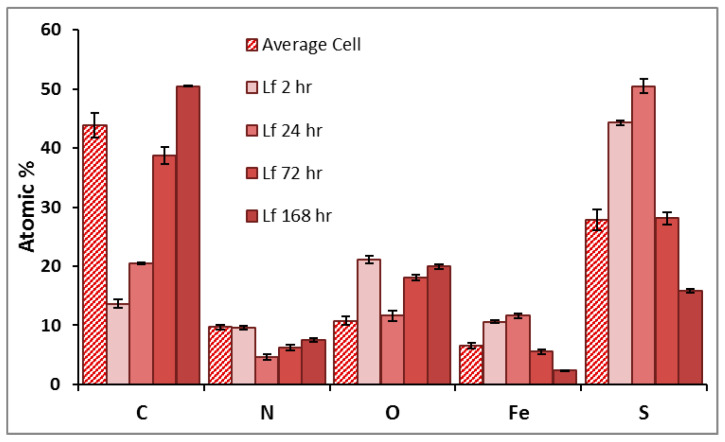
Average atomic percentage of pyrite exposed to *L. ferrooxidans* for 2, 24, 72, and 168 h, and the average atomic percentage of individual *L. ferrooxidans* cells (pattern), as determined by EDX.

The elemental composition of surface artifacts, and the identification of cellular material as distinct from secondary mineral precipitates was determined using EDX analysis. The EDX analysis of the pyrite surface exposed to *L. ferrooxidans* are shown in Figure 4 as average atomic percentage. The elements Si, Al, and Na were detected at levels below 2%.

The individual cells of *L. ferrooxidans* present a significant carbon signal compared to the bare spots mineral measurement; however, cells are typically not thick enough to block all signal from the bulk mineral beneath, which is why typical pyrite signals of Fe and S are still observed [58]. The pyrite surface exposed to *L. ferrooxidans* displays significant changes in elemental composition over time. Over the course of exposure, the amount of carbon, nitrogen, and oxygen detected on the surface increases, agreeing with the significant biofilm coverage of *L. ferrooxidans* on the surface, a phenomenon not observed in the *A. ferrooxidans* culture over this period.

**Figure 5:**
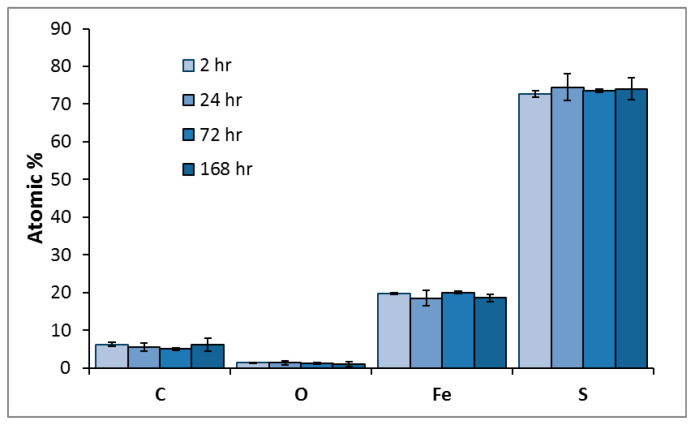
Average atomic percentage of pyrite exposed to HH medium for 2, 24, 72, and 168 h, as determined by EDX.

The EDX analysis of the pyrite surface exposed to HH medium are shown in Figure 5 as the average atomic percentage. The elements Al and Si were detected at levels below 2.5%. These results suggest the control surface is sulfur-rich, which agrees with the Eh results that show a greater concentration of iron leaching into the solution than sulfur, with no change occurring over the course of exposure.

As EDX penetrates the surface up to 5 μm, other experiments will be needed to investigate the outermost atomic layers with minimal bulk pyrite contribution and to relate the physical properties to chemical species on the surface.

### 3.3. Atomic Force Microscopy

As the topography of the surface has the potential to impact the wettability of a surface, the effects of surface leaching and pitting observed in the SEM images need to be isolated from the chemical changes on the mineral surface [59]. To assess the changes in the pyrite surface topography and isolate these effects from the chemical alterations brought about by *L. ferrooxidans*, atomic force microscopy (AFM) is used to quantify the physical impact of pyrite leaching.

**Figure 6:**
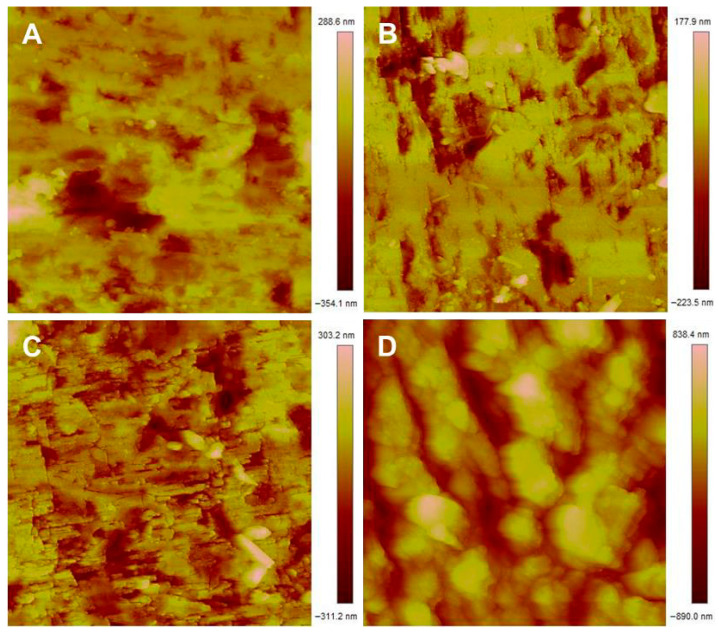
10 × 10 μm AFM height images of pyrite exposed to *L. ferrooxidans* for 2 h (**A**); 24 h (**B**); 72 h (**C**); 168 h (**D**).

Typical AFM images obtained for pyrite after different periods of exposure to *L. ferrooxidans* are shown in Figure 6. At least four images like those shown in Figure 6 were obtained per sample, always over an area of 10 μm, to calculate the average roughness of the sample.

**Figure 7:**
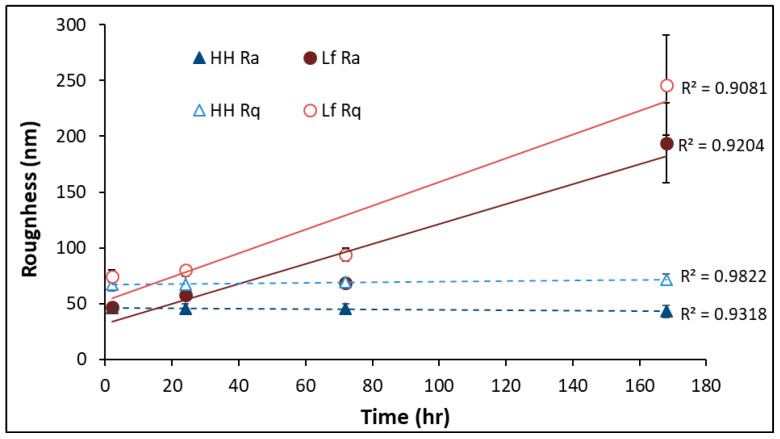
The roughness of pyrite exposed to *L. ferrooxidans* as calculated by *R_a_* (●) and *R_q_* (○), and HH medium as calculated by *R_a_* (▲) and *R_q_* (Δ).

The roughness of the pyrite surface exposed to *L. ferrooxidans* and HH medium as calculated by *R_a_* and *R_q_* are shown in Figure 7, with the error bars representing the 95% confidence interval.

The *R_a_* and *R_q_* of the pyrite surface increases linearly in the presence of bacterial cells, with correlation coefficients of 0.9204 and 0.9081, respectively. The increase in the standard deviation of samples over the course of exposure to *L. ferrooxidans* suggests there is more variation in roughness across the surface at advanced exposure times compared to those of shorter exposures. This agrees with the amount of uneven debris and pitting observed on the surface of the pyrite using SEM. The sample roughness of the pyrite exposed to *L. ferrooxidans* for 2 and 24 h does not differ significantly from the controls; however, after 72 h, the pyrite surface is quantifiably rougher after exposure to *L. ferrooxidans*. After 168 h of exposure, this difference in roughness is even more significant, agreeing with the initial observations made using SEM. The SEM images of bare polished pyrite are visually identical to those exposed to abiotic medium for 2 h. As was observed for *A. ferrooxidans*, the difference in roughness for the shortest periods of exposure to *L. ferrooxidans* and abiotic medium are statistically insignificant, and as such it can be assumed that the roughness of bare polished pyrite coincides with these values.

Previous studies that have applied AFM to studying *L. ferrooxidans* on the surface of pyrite have focused on cell morphology, sometimes coupling AFM with epifluorescence to observe cell adhesion and EPS production [23,25,28]. While more extensive studies have been performed on *A. ferrooxidans*, very few have investigated any cell preference for defect sites by *L. ferrooxidans*. Although preference for surface defects has been observed for other strains by previous investigations, this does not appear to be the case for *L. ferrooxidans* [32,60,61]. Other studies have suggested low crystallization areas have higher levels of ferrous iron and thiosulfate ions going into the solution [32,60]. This suggests that *L. ferrooxidans* would have ready access to iron nutrients on smooth areas of the surface.

The above results suggest that the presence of *L. ferrooxidans* is making the pyrite surface significantly rougher in comparison to the pyrite surface exposed to HH medium, and that this increase in roughness is proportional to the time spent in live culture. These results would suggest that the hydrophobicity of the HH medium pyrite control would be similar to pyrite exposed to *L. ferrooxidans* for short exposure times (less than 24 h), as their morphology over this period is not significantly different. These observations of the nature of the mineral surface must be compared to the measured hydrophobicity of the sample, to fully understand how *L. ferrooxidans* impacts the mineral surface in terms of its ability to depress or float pyrite.

### 3.4. Captive Bubble Contact Angle

**Figure 8:**
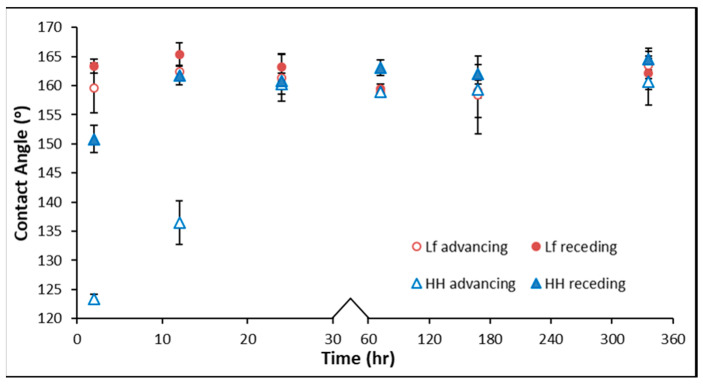
Advancing captive bubble contact angle of pyrite exposed to *L. ferrooxidans* (○) and HH medium (∆). Receding captive bubble contact angle of pyrite exposed to *L. ferrooxidans* (●) and HH medium (▲). Error bars represent sample standard deviation.

The measure of wettability by the captive bubble contact angle provides insight into the suitability of *L. ferrooxidans* as a potential flotation reagent. The contact angle provides an indication of how the microorganisms impact mineral wettability. Although some studies have measured the hydrophobicity of pyrite exposed to *A. ferrooxidans* [30], few have measured this aspect of pyrite exposed to *L. ferrooxidans*. Investigations into *L. ferrooxidans* in relation to mineral wettability have either measured mineral recovery by flotation in the presence of a synthetic collector or the contact angle of air-dried cells [28,35]. This leaves the impact of untreated cells on pyrite surface wettability unexplored. Figure 8 shows the average five advancing and receding contact angles of each pyrite sample, performed in triplicate, with the error bars representing the standard deviation of the samples measured.

Pyrite samples exposed to *L. ferrooxidans* are less hydrophobic than the HH medium control at early stages of exposure, showing the most significant decrease in hydrophobicity at 2 h of 12.5° to 36°. After 24 h, the pyrite exposed to *L. ferrooxidans* is still slightly less hydrophobic than the pyrite exposed to the HH medium, with advancing and receding angles greater by 3° and 25°, respectively. The pyrite exposed to *L. ferrooxidans* is more homogenous at early periods of exposure compared to the control, suggesting decreased hysteresis when cells are present. After 72 h, the contact angles are similar for both control and bacteria-exposed pyrite, with the contact angle appearing to remain stable for 336 h.

A difference in contact angle of as little as 3° can alter mineral recovery by up to 18%, depending on the size fraction of the particles [62]. These results suggest that an exposure of as little as 2 h could promote the depression of pyrite by *L. ferrooxidans*, while longer periods of exposure showed no significant separation of contact angle compared to the HH medium control sample. This suggests the hydrophobicity is improved by the chemistry on the surface rather than the roughness, which the AFM results suggest is identical between biotic and abiotic samples at early exposures. This also suggests the presence of a thick biofilm is not necessary for significant changes in the hydrophobicity of the pyrite, as the SEM showed little cell coverage after 2 h.

The biggest impact *L. ferrooxidans* has on the hydrophobicity of the pyrite in comparison to the HH medium control is in the early stages of exposure. At this stage, it is still not possible to determine whether the direct contact or indirect contact mechanism dominates the bacterial interaction with the pyrite surface. To separate physical changes on the surface such as roughness from potential chemical effects on the surface, surface chemical analysis must be performed on the system.

### 3.5. Scanning Transmission X-Ray Microscopy

Scanning Transmission X-ray Microscopy (STXM) is used on the fine particles of pyrite that are used to grow the bacteria in culture. This technique provides in situ physicochemical analysis of the ground mineral that would be necessary for flotation, an advantage over techniques that rely on flat and smooth samples that are less representative of flotation systems. As has been discussed, NEXAFS spectra provides information on the chemical bonding and oxidation states of the elements on the surface.

**Figure 9:**
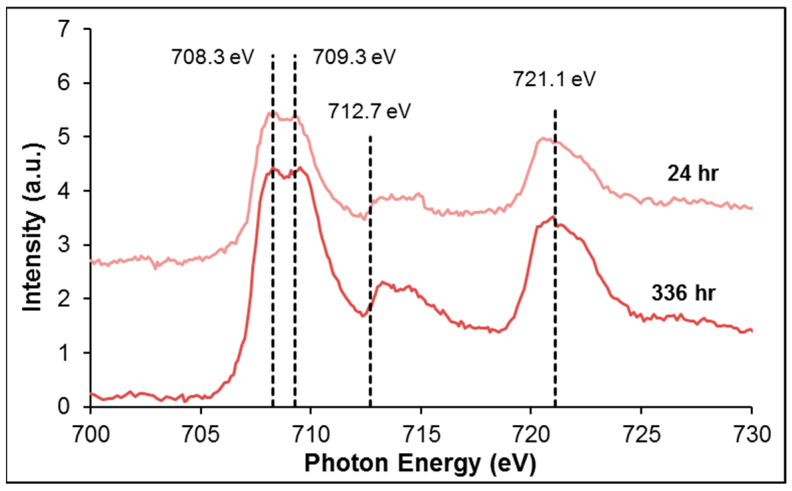
Fe L-edge NEXAFS spectra of pyrite exposed to *L. ferrooxidans* for 24 and 336 h. The transitions observed are to Fe 3*d* states hybridized with S 3*p* at 708.5 eV (overlapping with Fe 2*p* to Fe 3*d* states hybridized with O 2*p* states at 707.8–710.5 eV), S 3*p* states hybridized with Fe 4*s* and 4*p* states at 712–715 eV, and the Fe L_2_ peak at 719.9 eV.

The Fe L-edge NEXAFS spectra obtained for pyrite exposed to *L. ferrooxidans* for 24 and 336 h are shown in Figure 9. The samples were prepared by drop casting and air drying onto a Si nitride window. The behavior of pyrite exposed to the HH medium control solution was investigated using Fe L-edge NEXAFS in previous work, and showed no significant change over time [30].

The transitions that cause the peaks present in the pyrite Fe L-edge NEXAFS spectra have been described previously [46,63,64]. The spectrum for pyrite exposed to *L. ferrooxidans* for 336 h shows the Fe L_3_ transitions corresponding to the Fe 3*d* states hybridized with S 3*p* and iron oxides and (oxy)hydroxides at 707.9–708.8 eV [46,63,64,65], and the S 3*p* to Fe 4*s* and 4*p* transitions at 712–715 eV [45,66]. The iron oxidation products occurring on the surface obscures the bulk pyrite signal. The increased surface area of particles compared to flat coupons is likely to be at least partially responsible for this oxidation. The NEXAFS spectra obtained for pyrite shown above agree heavily with those obtained by Goh et al. [45], and generally agree with studies that used synthetic pyrite or pyrite samples that were not exposed to air, although they did not have the resolution to observe the two overlapping peaks, they infer the existence of the structure seen by Goh et al. and in this study [46,63].

**Figure 10:**
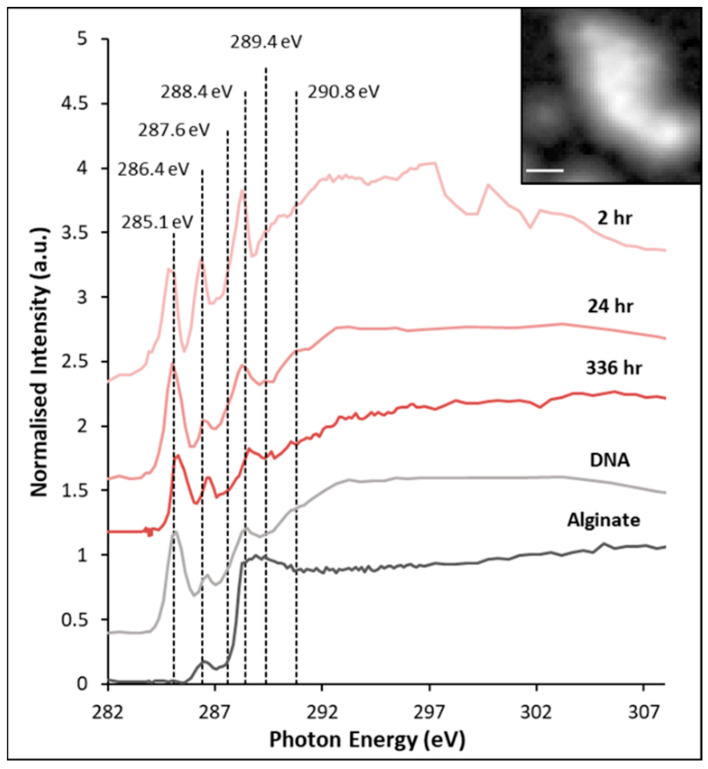
C K-edge NEXAFS spectra of chalcopyrite exposed to *L. ferrooxidans* for 2, 24, and 336 h, bovine serum albumin (DNA) and sodium alginate (used for calibration) Inset: carbon image stack (scale 1 μm). The transitions observed on pyrite exposed to *L. ferrooxidans* are from C 1s to π* C=C and C-H at 285.1 eV, π* C=N and π* C=C at 286.4 eV, π* C=O at 288.4 eV, σ* CNH, σ* CH or π* C=N at 289.4 eV, and σ* C-C at 292–294 eV.

The behavior of pyrite exposed to the HH medium control solution was investigated using C K-edge NEXAFS in previous work, and showed no significant change over time [30]. Briefly, the signals indicated potential radiation damage [67,68], and the presence of adventitious carbon [69,70]. Figure 10 shows the stacked C K-edge NEXAFS spectra of pyrite exposed to *L. ferrooxidans* for 2, 24, and 336 h, and the changes in the carbon species occurring over those exposure times, as well as both DNA and sodium alginate standard spectra. All spectra were corrected to the 285.1 eV of the aromatic π* C=C peak of DNA.

**Figure 11:**
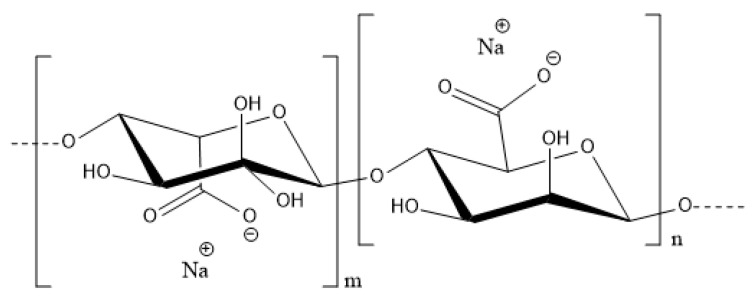
Chemical structure of sodium alginate.

The C K-edge NEXAFS spectra of pyrite exposed to *L. ferrooxidans* were collected over the total area of the carbon stack image, an example of which is shown above (Figure 10, inset). The NEXAFS spectra were collected over an area of the sample to improve the signal, which was very weak. The spectra collected of DNA and sodium alginate display peaks in expected areas, with sodium alginate not displaying signal at 285.1 eV due to no aromatic C=C functionalities occurring within its structure, shown in Figure 11.

Every C K-edge NEXAFS spectra each of the pyrite samples exposed to *L. ferrooxidans* is showing the π* C=C peak at 285 eV, and second intense signals at 286.4 eV, which have been associated with both π* C=N and π* C=C peaks [47,48,71,72,73,74,75]. All spectra are also showing broad signal in the 292–294 eV region that can be attributed to σ* C-C [47,48,75]. The π* C=O peak after 2 h occurs between 288.4 and 288.7 eV, suggesting that the peak is due to more carboxylic-type character common in lipids and biopolymer [47,71,72,76,77]. After 24 h, the π* C=O peak appears broader, suggesting an increase in signal due to the π* C=O bonds in amides of protein, which typically occur at 288.2 eV [47,48,72,73,75,76,78]. The protein-type π* C=O peak is associated with the increase in the peak at 289.4 eV, which can be attributed to σ* CNH, σ* CH or π* C=N amide functionality in proteins and nucleic acids [47,48,78]. After 336 h, the protein contributions have decreased, while the π*C=O at 288.4 eV of polysaccharides remains strong. The π* C=C peak at 285.1 eV may also be indicative of radiation damage as C-H and C-C bonds are reduced; however, as the sodium alginate spectrum is displaying no signal in this area, radiation damage appears to be unlikely in these samples [67,68].

Several papers have previously studied bioleaching of minerals using *L. ferrooxidans*, although many of them investigating the mineral surface use NEXAFS, as the investigation of carbon of the cells themselves is challenging in most UHV techniques [27,79,80]. Other studies have used the STXM technique to collect carbon NEXAFS of other types of cells, and although they have identified similar functionalities, the spectra do not have the same shape, indicating a different EPS composition to *L. ferrooxidans* [47,76,81].

These samples provide evidence for why the difference in hydrophobicity of pyrite exposed to *L. ferrooxidans* at 2 h is decreased compared to the sample exposed to the HH medium control; hydrophilic polysaccharide-type species [35,82,83] produced by the bacteria are detected at this stage of exposure, creating a less hydrophobic surface compared to the pyrite exposed to HH medium. As cells start to colonize the surface and oxidation of the mineral progresses, the sample becomes slightly less hydrophobic after 24 h compared to the control. After 336 h of exposure to *L. ferrooxidans*, the pyrite surface appears to be slightly more oxidized than that at 24 h, and there seems to be little change in biofilm composition between 24 and 336 h. Anionic functional groups in EPS components are present at early stages of exposure, providing binding sites for iron and promoting pyrite oxidation without the need for cell attachment to the surface [84,85]. Due to the presence of iron-complexing EPS on the pyrite surface, it is likely that the indirect contact mechanism most accurately describes the interaction of *L. ferrooxidans* with pyrite over the early periods of exposure [29,86,87].

These changes do not correlate with the AFM results, which suggest that the hydrophobicity of the pyrite exposed to *L. ferrooxidans* should be greater than the hydrophobicity of the control, as correlates with the roughness increasing [59]. These results suggest that the effects of the chemical species on the surface are greater than those of the physical changes to the surface brought about by the bacteria. This indicates the indirect contact mechanism, which relies on EPS for the alteration of the mineral surface, may dominate the interaction of *L. ferrooxidans* with pyrite.

### 3.6. Time of Flight–Secondary Ion Mass Spectrometry

ToF-SIMS provides chemical and elemental information on the outermost atomic layers of the surface, providing semi-quantitative information on the chemistry contributing to the hydrophobicity of the mineral surface compared to X-ray techniques that penetrate the surface. Although individual compounds in a mixture are not yet able to be individually identified, a “fingerprint” can be obtained of the fragments identified on the surface of the pyrite exposed to *L. ferrooxidans*, which may lead to the identification of the types of molecules on the surface that impact contact angle [69,88,89,90].

**Figure 12:**
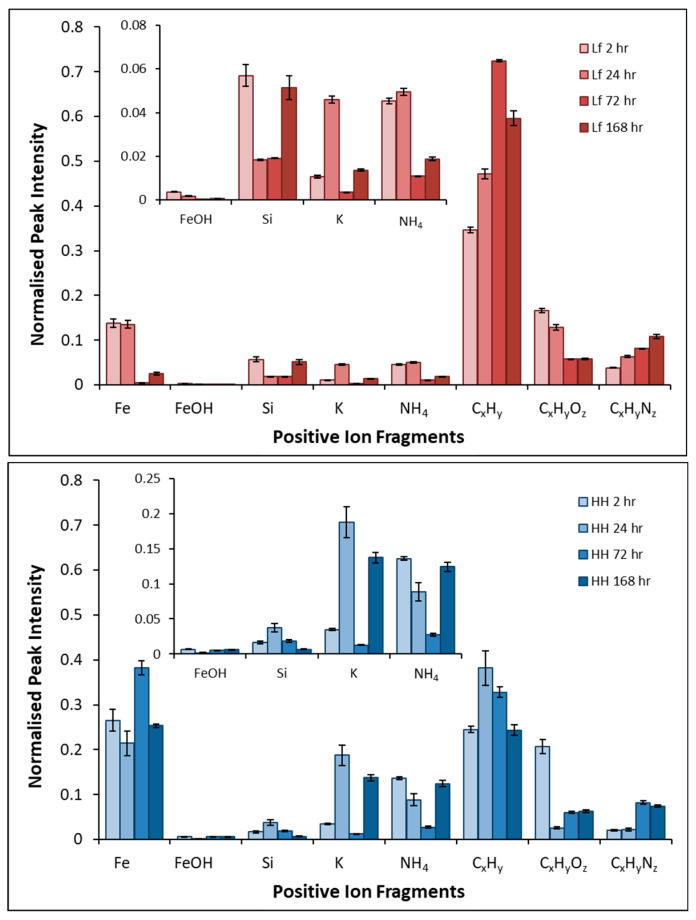
Average normalized peak intensities of positive fragments of pyrite exposed to *L. ferrooxidans* (**top**) for 2, 24, 72, and 168 h, and pyrite exposed to HH medium (**bottom**) for 2, 24, 72, and 168 h.

The plots shown here are an indication of the proportion of each fragment on the mineral surface, and are only comparable with other fragments of the same sample. Figure 12 shows the positive ions on the surface of pyrite exposed to *L. ferrooxidans* and HH medium for 2, 24, 72, and 168 h.

The fragment profile of pyrite exposed to *L. ferrooxidans* is dominated by short chain C_x_H_y_^+^ (x ≤ 6) fragments for every period of exposure, with the C_x_H_y_O_z_^+^ (x ≤ 4, z ≤ 2) fragments in high proportions at early periods of exposure. The high proportion of hydrophilic C_x_H_y_^+^ and C_x_H_y_O_z_^+^ fragments at these early periods of exposure compared to the more iron-rich control samples suggest that the *L. ferrooxidans* cells are producing polysaccharides, making the surface less hydrophobic [35,82,83]. This agrees with the results presented in both the STXM and contact angle studies that suggest the presence of polysaccharides during early periods of exposure, making the pyrite surface significantly less hydrophobic than the control.

After longer periods of exposure, the proportion of C_x_H_y_N_z_^+^ (x ≤ 5, z ≤ 2) fragments increases significantly. The increase in the proportion of hydrophobic protein-type signals is likely to be partially responsible for the increase in hydrophobicity observed at this point in the contact angle results. These results also suggest that *L. ferrooxidans* produces more protein-type compounds as biofilm begins to build up on the mineral surface as was observed by SEM. However, as *L. ferrooxidans* is a known EPS producer, the proportion of organic fragments observed on the pyrite surface upon exposure may be reasonably expected to be significant enough to cause the decreased hydrophobicity of the pyrite after short periods of exposure [17,23,32].

The proportion of Fe^+^ detected on the surface decreases over the course of exposure, suggesting the layer of bacterial cells that was shown to spread over time is obscuring the mineral below, resulting in the dramatic decrease in proportion. Due to medium salt precipitates on the surface, both NH_4_^+^ and K^+^ are expected to be detected on the surface. These salt precipitates mean the NH_4_^+^ fragment is not a reliable indicator of proteins on the surface. There are very small amounts of Si^+^ and FeOH^+^ on the surface, indicating little to no jarosite formation on the surface and some silicate inclusions. The proportions of these fragments remain low over the course of exposure. This agrees with the NEXAFS results that also suggested little jarosite or secondary minerals formation from the Fe L-edge spectra.

**Figure 13:**
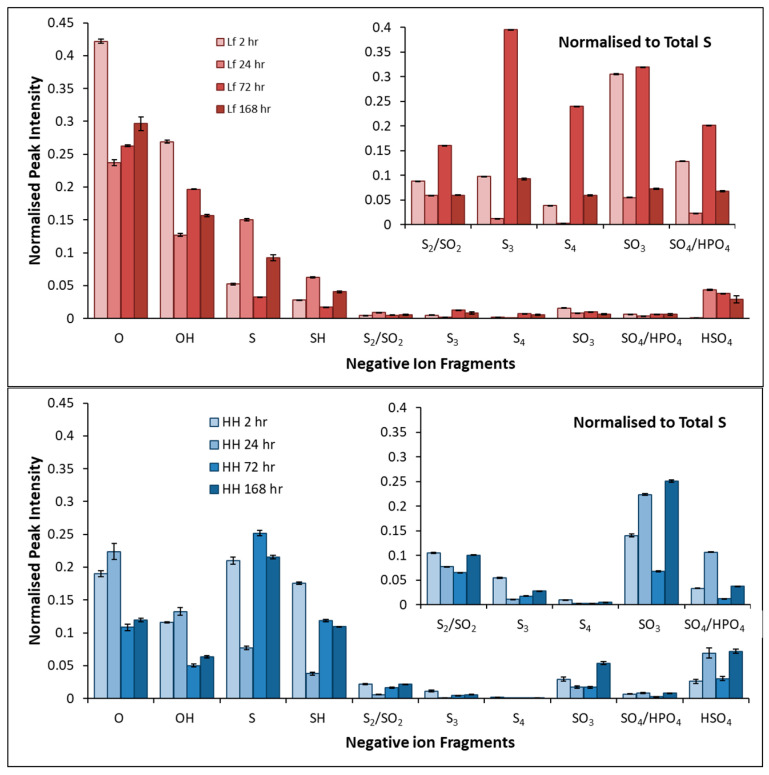
Average normalized peak intensities of negative fragments of pyrite exposed to *L. ferrooxidans* (**top**) for 2, 24, 72, and 168 h, and pyrite exposed to HH medium (**bottom**) for 2, 24, 72, and 168 h.

Figure 13 shows the complementary negative fragments and elements of interest and significance collected by ToF-SIMS on pyrite exposed to *L. ferrooxidans* and HH medium for 2, 24, 72, and 168 h. The inset plots in Figure 13 show the sulfur species on the surface normalized to the total S^−^ detected on the surface, showing the proportion due to each sulfur species. The fragments detected on pyrite after exposure to *L. ferrooxidans*, are dominated by the O^−^ and OH^−^ fragments for all samples. Over earlier periods of exposure, the proportion of hydrophobic sulfur species is much lower compared to the control. This agrees with the contact angle data results that show the pyrite exposed to *L. ferrooxidans* is less hydrophobic than the control after 2 h. Oxygenated sulfur species such as SO_3_^−^ and SO_4_^−^ are in greater proportion on the surface than polysulfide species such as S_3_^−^ and S_4_^−^, although the overall proportion of sulfur species on the surface is much smaller than that observed on the control pyrite for the duration of exposure.

Over longer periods of exposure the proportion of polysulfide species on the pyrite exposed to *L. ferrooxidans* increases, with longer periods of exposure resulting in larger proportions of polysulfide on the surface than the control for the same periods. This increase in polysulfides coincides with an increase in ferric iron in the solution, indicating the surface has become more sulfur-rich as iron is leached from the surface of the mineral and consumed by the bacteria. This agrees with the contact angle results that show a slight increase in hydrophobicity of the pyrite over 72 h of exposure to *L. ferrooxidans*; however, as biofilm grows, the surface remains hydrophilic overall.

**Figure 14:**
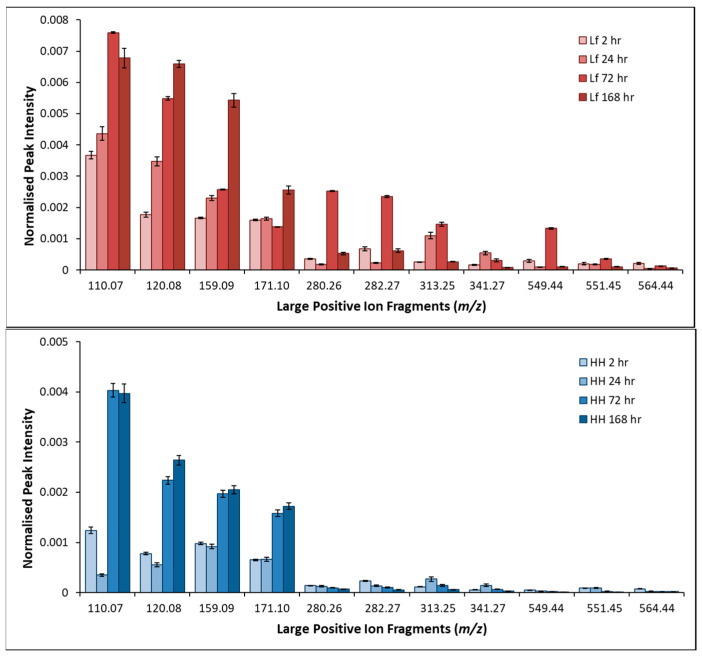
Average normalized intensity of large positive fragments of pyrite exposed to HH medium (**top**) and *L. ferrooxidans* (**bottom**) 2, 24, 72, and 168 h.

Larger molecular weight fragments associated with the production of EPS were detected on the surface of pyrite exposed to *L. ferrooxidans*. Figure 14 shows the large molecular mass positive fragments collected by ToF-SIMS on pyrite exposed to *L. ferrooxidans* and HH medium for 2, 24, 72, and 168 h.

Initially, there are multiple large molecular weight fragments on the pyrite surface after exposure to *L. ferrooxidans*, that increase significantly by 168 h. The fragments that follow this increasing frequency trend are of *m*/*z* 110.07, 120.08, and 159.09 and have been identified as amino acid fragments by several previous studies, which may indicate DNA or protein on the surface increasing in frequency over time as biofilm increases on the surface [89,91,92,93,94,95]. The remaining fragments have variously been identified as major components of several lipid and carbohydrate structures [88,90,95,96,97,98,99]. The fragment with *m/z* 280.26 follows the same trend as other polysaccharide-type fragments, indicating that it is also likely to be a large polysaccharide fragment, although the accurate identity is not possible to ascertain. The proportion of these fragments are significantly greater in pyrite samples exposed to *L. ferrooxidans* compared to the pyrite exposed to HH medium, with the polysaccharide-type fragments having larger proportions at earlier exposure times, with the amino-acid fragments becoming more frequent at 72–168 h. The occurrence of these fragments indicates that large hydrophilic polysaccharide and lipid fragments are present and being produced very shortly after inoculation, which is consistent with both the NEXAFS and contact angle results presented in previous sections. These results support the indirect contact mechanism as describing the interaction of *L. ferrooxidans* with pyrite over early periods of exposure; however, the build-up of biofilm and the increase in high mass fragments due to EPS components and cellular material make it impossible to determine whether the direct or indirect contact mechanism dominates in longer periods of exposure.

**Figure 15:**
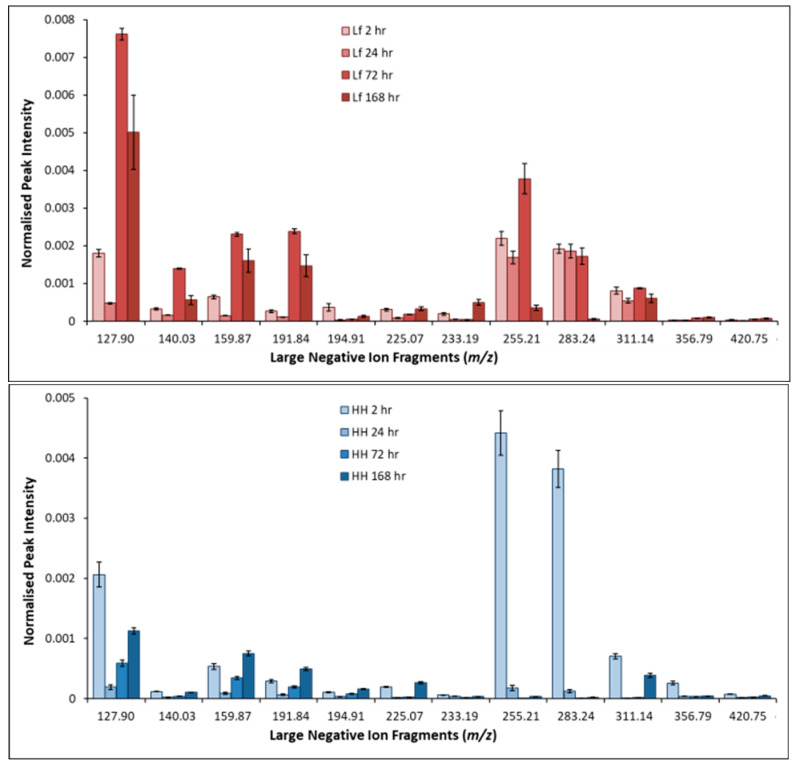
Average normalized intensity of large negative fragments of pyrite exposed to HH medium (**top**) and *L. ferrooxidans* (**bottom**) 2, 24, 72, and 168 h.

The negative ion fragments of large molecular weight are less well understood than the positive fragments that can be used to compliment the positive ion spectra. Unique EPS fragments can also be identified in the negative ion spectra. The average normalized peak intensity of large molecular weight negative fragments of pyrite exposed to *L. ferrooxidans* and HH medium for 2, 24, 72, and 168 h is shown in Figure 15.

Large molecular weight negative fragments are not explored as extensively as positive fragments, but the those observed in this study support the conclusions of the positive ion fragments, indicating larger molecular weight molecules are present in early stages of attachment in statistically significant amounts and decrease as exposure continues. The fragments with *m/z* 127.90, 140.03, 194.91, 255.23, 283.25, and 311.14 have both been identified as fatty acid or carbohydrate fragments in previous studies [96,97,98,100,101]. As the fragments at *m/z* 159.87, 191.84, 225.07, 233.19, 356.79, and 420.75 follow the same trend, it is not unreasonable to suggest that these fragments are also due to polysaccharides and/or lipids.

Previous investigations of pyrite surfaces using ToF-SIMS were focused mainly on identifying pyrite particles within a mixture of sulfide minerals, and on the ions associated with the pyrite surface and ions of interest to flotation. The results presented here for the control pyrite are much in agreement with previous studies that have observed, under a wide range of conditions, that the surface of pyrite is iron-rich; with hydrophilic iron hydroxides leading to pyrite depression while hydrophobic sulfur-rich surfaces lead to flotation [102,103,104,105,106].

Previous studies that have utilized ToF-SIMS for the investigation of microorganisms and their excretions have mainly focused on the separation of known components such as proteins and monosaccharides by principle component analysis; however, it has yet to be successfully applied to complex samples [89,107,108]. Our results agree with those obtained by Pradier et al., who observed that the negative spectra of bacteria are dominated by the O^−^ and OH^−^ fragments, which agrees with the strong overlap of those ions with hydrocarbons caused by bacteria observed here [109]. They also found bacterial species rich in carbohydrates and proteins, which they concluded by summing oxygenated and nitrogenated carbon signals, respectively, achieved the highest adhesion to steel surfaces, with carbohydrate rich cells adhering most effectively [109]. Other studies have looked at specific types of compounds such as proteins or peptides on cells, and Dague et al. specifically related protein fragments as being hydrophobic, a contribution which is also observed here [91,95].

## 4. Conclusions

It was observed using SEM that attachment of cells occurs at very early stages of exposure, with no obvious preference for surface defects, and that this initial attachment develops into uniform, monolayered biofilm by 168 h of exposure. Leaching of the pyrite is accelerated by the presence of *L. ferrooxidans*, with the roughness of the surface as measured by AFM confirming advanced pitting compared to the acidity in the medium alone. The difference in hydrophobicity is greatest between bacterial exposure and abiotic control at the early exposure stages of 2 h, which coincides with the presence of polysaccharide and fatty acid-type structures measured by both NEXAFS and ToF-SIMS. Longer periods of exposure lead to inorganic oxidation of the pyrite surface exposed to HH medium, and increased production of hydrophobic proteins and sulfur-rich passivation layers on pyrite exposed to cells, causing little difference in hydrophobicity of the samples by the time full biofilm forms at 168 h.

These observations suggest that *L. ferrooxidans* preferentially produces polysaccharide and fatty acid compounds to assist with initial adhesion to pyrite, before beginning to produce more hydrophobic proteins as colonies begin to develop on the surface. They also produce significantly more of these products that the *A. ferrooxidans* studied previously [30], providing a greater difference in hydrophobicity compared to pyrite exposed to HH medium. These results suggest both mesophiles interact through the indirect contact mechanism during early periods of exposure to the surface of pyrite. This has important implications for the field of bioflotation, which would ideally require short-term exposure for the most efficient separation of minerals and a larger amount of the product required to affect the desired changes. This study suggests that not only is the nature of bacterial excretions changing, but that similar changes may be occurring across strains and that strains that produce more EPS are potentially more efficient at providing the means of mineral separation.

## Data Availability

The original contributions presented in this study are included in the article. Further inquiries can be directed to the corresponding author.
